# The Usage Effects, Effect Modifiers, and Experiences of a Web-Based App for Healthy Habit Formation in Adults: Exploratory Analysis of a Quasi-Experimental Real-World Intervention

**DOI:** 10.2196/84076

**Published:** 2026-09-21

**Authors:** Eeva Rantala, Mikko Valtanen, Adil Umer, Suvi Parikka, Jussi Pihlajamäki, Ilona Ruotsalainen, Jaana Lindström

**Affiliations:** 1 Department of Public Health, Finnish Institute for Health and Welfare, P.O. Box 30, Helsinki, 00271, Finland, 358 (0)29 524 7224; 2 Department of Mathematics and Statistics, University of Turku, Turku, Finland; 3 Critical cyber-physical systems team, VTT Technical Research Centre of Finland Ltd., Tampere, Finland; 4 Institute of Public Health and Clinical Nutrition, University of Eastern Finland, Kuopio, Finland; 5 Department of Medicine, Endocrinology and Clinical Nutrition, Kuopio University Hospital, Kuopio, Finland; 6 Health Data Analytics team, VTT Technical Research Centre of Finland Ltd., Kuopio, Finland

**Keywords:** web-based, app, lifestyle, habit, behavior change, diet, physical activity, BMI, health promotion, prevention

## Abstract

**Background:**

Digital interventions provide a scalable approach to promote healthy lifestyles, but at-scale evidence of their effects and effect modifiers is limited—even scarcer for habit-based digital interventions, although habits are promising lifestyle intervention targets.

**Objective:**

We conducted a 1-group pre-post effect and effect modification assessment of a web-based app designed to support the formation of health-promoting lifestyle habits by exploring the relationship between objective app use and subjective changes in behavioral outcomes.

**Methods:**

Three-month app access was offered to a subsample of the population-based Healthy Finland survey participants aged 20-74 years via SMS text messaging or mail. The app provided personalized behavioral suggestions that translated evidence-based lifestyle guidelines into simple, repeatable actions (“habits”). Users could browse and select these habits, and report and monitor their performances. The assessment used app log data, questionnaires collected through the app at baseline and at 45 and 90 days, and background information obtained from the national population register and the Healthy Finland Survey. Linear mixed effects models explored the (1) associations between app use and changes in self-reported diet quality (Healthy Diet Index), physical activity (metabolic equivalent of task hours per week), and BMI, and (2) modification of these associations by baseline characteristics related to sociodemographics, health, lifestyle, and e-service use. App use measures comprised the percentage of app use days (ie, days with logins/days of follow-up×100) and the number of reported habit performances (total, diet-related, physical activity–related, and BMI-related).

**Results:**

Of 6975 invitees, 1282 (18.4%) accepted the invitation and 382 (5.5%; mean age 51, SD 15 y; women: n=263, 69%) completed the data collection required for the assessment. Over 90 days, the median percentage of app use days was 5.9% (IQR 3.3%‐11%), and the total number of reported habit performances was 22 (IQR 3‐68.5). A higher percentage of use days (by 10 percentage points) was associated with a 3.09 (95% CI 0.79‐5.39) metabolic equivalent of task hours per week greater increase in physical activity and a 1-unit higher logarithmic number of reported performances with a 0.51 (95% CI 0.07‐0.95) Healthy Diet Index point greater improvement in diet quality. Other associations between app use and outcomes were nonsignificant. Greater physical activity and a more positive attitude to e-services at baseline appeared to enhance the effect of app use on physical activity (*P* values <.05). The app received a median acceptability rating of 2.9 (IQR 2.4‐3.3, scale: 1‐4) and a median overall score of 7 (IQR 5‐8, scale: 0‐10).

**Conclusions:**

An inexpensive, low-intensity digital support for healthy habits could foster small beneficial lifestyle changes, but effects require user engagement and may depend on the individual. The findings warrant confirmation in more robust study designs and call for further research for identifying those most likely to benefit from such digital support.

## Introduction

Digital lifestyle interventions are considered a relatively inexpensive and scalable way to support the adoption and maintenance of healthy lifestyle behaviors. They enable a delivery independent of time, place, and health professionals and provide a promising avenue to increase the availability and accessibility of health services without additional burden on the health care system. Emerging evidence also suggests that digital lifestyle interventions can save societal and health care costs [1,2]. They thus appear to be an attractive solution for aging societies that face an increasing need for care while under pressure to reduce health care expenses.

While literature reviews have drawn varying conclusions on the overall effectiveness of digital lifestyle interventions, they have demonstrated that diverse digital tools have the potential to improve health behaviors and other health outcomes among various adult populations [3-10]. For example, a recent umbrella review and meta-meta-analysis of 47 systematic reviews covering more than 500 unique randomized controlled trials (RCTs) and more than 200,000 participants found grade A–level evidence that digital lifestyle interventions (mobile apps, web-based, SMS text messaging, or mixed) yield, on average, small to medium favorable effects on dietary outcomes (fruit, vegetable, and energy intake), physical activity (total, moderate to vigorous, and steps per day), and body weight when used predominantly as stand-alone treatments [4].

However, lifestyle change can be promoted in a multitude of ways. Habit-based approaches focus on the repetition of a specific behavior in a consistent context [11,12]. These approaches promote the formation of habitual behaviors that occur automatically as a response to specific contextual cues—even without conscious deliberation, motivation, or awareness [11-13]. Supporting habit formation has the potential to result in lasting lifestyle change, which sustains even when motivation drops [12]. An umbrella review with meta-meta-analyses of behavior change interventions across behavioral domains found habits to be the most potential targets of individual-level interventions, compared with knowledge, beliefs, attitudes, skills, and emotions [14]. For weight loss, nondigital habit-based lifestyle interventions have yielded evidence of short-term effectiveness compared to no treatment or usual care among individuals with overweight or obesity [15].

Nevertheless, the habit-based approach has not yet been widely adopted in digital lifestyle interventions. Furthermore, the few digital solutions that have used a habit-based approach have mainly been studied in specific population groups or in small-scale trials. Examples include the HabitWalk app for increased brisk walking that was tested in a 15-week microrandomized trial among inactive adults [16] and the Top Tips app for weight loss that was piloted in a 3-month RCT among adults with overweight or obesity [17]. Another example was the BitHabit app that was developed for the prevention of type 2 diabetes (T2D) and tested in a 12-month RCT among adults with T2D risk factors [18,19]. While the HabitWalk study demonstrated that the repetition of a target behavior in response to a specific cue is positively associated with habit strength [16], both the Top Tips and BitHabit apps showed the greatest effects among study participants who engaged with the apps more frequently [17,20]. These results indicate that habit-based lifestyle apps may be beneficial as long as they are used and highlight the importance of incorporating use metrics in their effect assessments. Sustained engagement remains a key challenge in digital lifestyle interventions [3,4,16,17,21,22]. The learnings of habit-based and other lifestyle apps suggest that personalization could enhance engagement [17,23,24].

Overall, limited evidence exists of the effects of digital lifestyle interventions—habit-based and other—among the general adult population and thus the potential of these interventions to promote public health at scale. Similarly, evidence of individual factors that may modify the effects of digital lifestyle interventions is limited and inconclusive [3,4,7,23,25]. Studying such effect modifiers is a prerequisite for identifying population groups that could benefit from exclusively digital interventions and groups that would need other types of support, such as in-person counseling.

This study addresses the knowledge gaps mentioned earlier regarding the effects and effect modifiers of digital lifestyle interventions intended to support the formation of health-promoting habits among the general adult population. Simultaneously, the study serves as a real-world example of scaling up a habit-based lifestyle app originally developed for individuals at risk of lifestyle-related chronic noncommunicable diseases to a more heterogeneous adult population. The study used a quasi-experimental pre-post design and deployed the app as a stand-alone support for self-directed habit formation. The overall aim was to conduct an exploratory assessment of the effects of the app use on behavioral outcomes and to identify groups most likely to benefit from using the app. The specific aims were to examine (1) associations between objectively measured app use and changes in self-reported diet quality, physical activity, and BMI over 3 months; (2) the modification of these associations by the baseline characteristics of the participants; and (3) the participants’ experiences (ie, acceptability and overall evaluation) of the app.

## Methods

### Study Design

This study followed a quasi-experimental 1-group pretest-posttest design in which the participants were followed up for 3 months via the app using continuous app log data and questionnaires conducted at baseline, midway through (approximately 45 days since the baseline), and at the end of the follow-up (approximately 90 days since the baseline). The effects and effect modifiers of the app were examined based on an exploratory analysis of the relationship between objectively measured app use and changes in self-reported behavioral outcomes. Owing to the correlational nature of the assessment, the reporting of the study follows the STROBE (Strengthening the Reporting of Observational Studies in Epidemiology) guideline for cohort studies [26,27] (for a completed checklist, see Checklist 1).

### Ethical Considerations

The research plan of the study was approved by the Institutional Review Board of the Finnish Institute for Health and Welfare (THL/5335/6.02.01/2022). The study participants gave their informed consent to participate. Participation was free of charge, and participants were not compensated.

### Participant Recruitment

This study was a substudy of a population-based health survey, Healthy Finland, that targeted a randomly selected and nationally representative sample of adults in Finland [28]. Participants of the Healthy Finland survey were eligible for this study if they (1) spoke Finnish fluently, (2) were aged 20 to 74 years, (3) had completed the questionnaire of the Healthy Finland survey between September and the end of the year 2022, and (4) were not randomly selected for an additional health examination component of the Healthy Finland survey. The latter criterion was to avoid influencing the health examination results and to avoid burdening the participants excessively. Participation in this study also required access to the internet via a smartphone, tablet, or computer.

Among the eligible population (N=10,207), individuals with a known phone number (n=4978, 48.8%) were sent a personal invitation to participate in this study in February 2023 via SMS text message. From the remaining eligible population, 2000 individuals (19.6% of the 10,207 eligible people) were sampled based on 5-year age groups to receive the invitation via letter. The sample size was determined for the assessment of the uptake of the app [29,30] and estimated to be sufficient to detect small effects based on logistic regression with a statistical power of 0.8 and a type I error of 0.05, assuming that 10% to 15% of the invitees would choose to participate. The invitation was valid until the beginning of March 2023. The invitation directed the participant to a website of the Finnish Institute for Health and Welfare, which provided information on the study and the app, a link to the app, and instructions on how to start using the app. Of the 6975 invitees (excluding 3 who asked for their data to be removed), 1282 (18.4%) accepted the invitation, agreed to the terms of the app, and registered for the app with their phone number and a user ID that was featured in the invitation [29,30].

### The App

The app used in this study was web-based and closed (ie, not open access) and could be used with a smartphone, tablet, or computer with no need to download the app on the device. The app was based on the BitHabit app that was developed in a T2D prevention study, Stop Diabetes (StopDia) [18,19,31], which involved the authors of this study and/or their affiliated research institutes. The BitHabit app was designed to support the adoption of lifestyle habits that promote health and aid the prevention of T2D and other lifestyle-related chronic diseases [19,20]. The BitHabit app was built on the principles of habit formation [32-34] and self-determination [35,36] theories. Habit formation approaches promote the repetition of a target behavior in the presence of consistent contextual cues until the cues begin to trigger the behavior automatically [32-34]. Self-determination theory emphasizes the role of autonomous motivation, perceived competence, and a sense of community in lifestyle changes and fosters the fulfillment of basic psychological needs—the perception of autonomy, the sense of control or self-efficacy, and the feeling of relatedness with peers or significant others [35,36]. After the StopDia study, the BitHabit app was updated by improving its visual appearance and by adding features for personalization (habit suggestions based on a background questionnaire) and for social support (a researcher-moderated discussion forum). This updated version was used in this study and is referred to as “the app” because the BitHabit brand has been out-licensed.

The app used in this study provided an “online store” or “library” of approximately 300 behavioral suggestions that translated lifestyle guidelines [37-41] into concrete actions. These actions were simple, specific, contextualized, and easily repeatable, and they were presented with a brief description of how they promote health. The actions were tied to and could be triggered by daily life activities, which made them optimal for habit formation [19]. The actions were organized into 19 habit categories related to eating and drinking (10 categories: mindful eating, meal pattern, vegetarian and fish courses, fruits and vegetables, grain products, dietary fats, sugar, salt, alcohol, and drinking water), physical activity (5 categories: aerobic exercise, muscle strengthening exercise, mobility and balance activities, stretching one’s legs, and everyday physical activity), sleep, positive mood, daily life management, and smoking.

**Figure 1.**
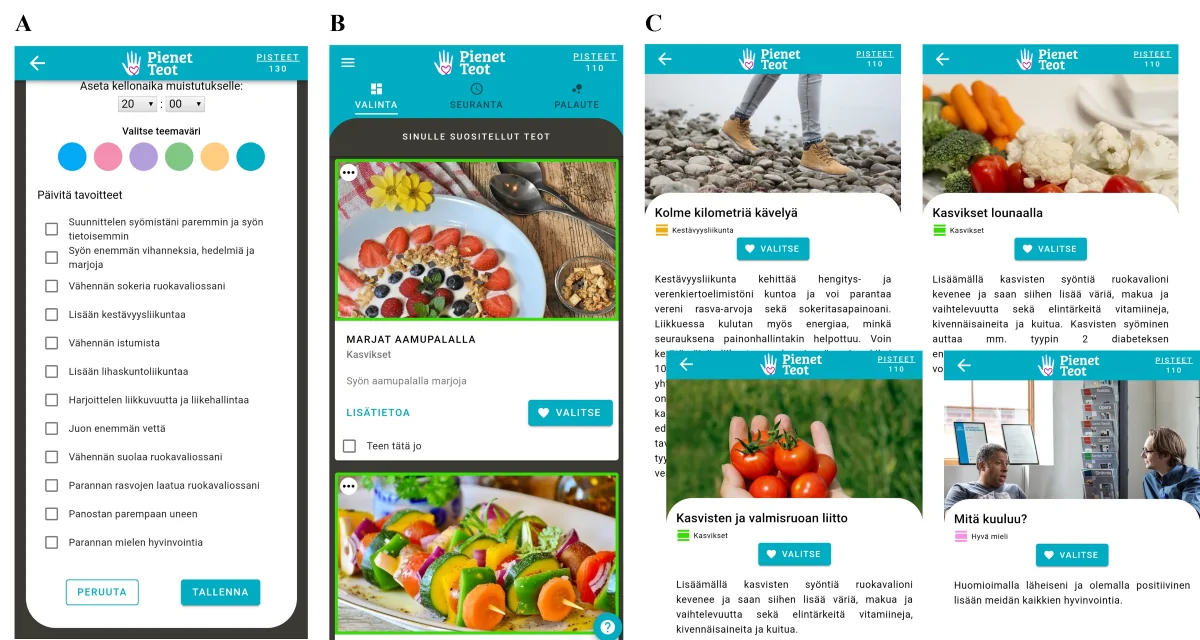
Views of the app that (A) allowed setting well-being goals, reminders, and a theme color; (B) provided habit suggestions based on the goals set; and (C) described specific habits.

On their first login to the app, the participants of this study gave their informed consent and completed a brief questionnaire on personal characteristics (eg, age, sex, and employment or study situation), health behavior (dietary habits, alcohol consumption, physical activity, sleep, and smoking), perceived stress, and well-being goals. On the basis of the participant’s responses to the questionnaire, the app determined the habit categories with the greatest potential for improvement and suggested the categories accordingly. After this, the participant could enter the app to browse the habit library; to inspect, select, and report actions; and to monitor and reflect on the actions performed. In addition, the app allowed setting reminders, enabled modifying the reported well-being goals, provided summary data on actions performed by other users, featured a researcher-moderated forum for discussion and questions, and provided information on health-promoting lifestyles. If the participant did not use the app for over a week, they automatically received up to 3 SMS text message reminders with 1-week intervals. No adjustments were made to the content or functionalities of the app during the study follow-up. Figure 1 illustrates the visual appearance and functionalities of the app.

### Measures

#### Participant Characteristics

The participants’ age and sex (male or female, as assigned at birth) were retrieved from the Finnish National Population Register. Information on socioeconomic status, health, lifestyles, and the use of e-services was obtained from the questionnaire of the Healthy Finland survey, which the participants had completed a median of 148 (range 47‐231) days before the baseline of this study.

Information on socioeconomic status comprised education (years of full-time schooling, including primary and comprehensive school), household income (euros per month before taxes) as a 5-category variable, and principal life situation (employed or studying, on family leave or a stay-at-home parent, retired, unemployed, or other). Health-related information comprised perceived health status (poor or fairly poor vs average, fairly good, or good) and perceived cognitive function (average of 3 items: the working of memory, ability to learn new things, and ability to concentrate). Lifestyle-related information comprised getting enough sleep (rarely or hardly ever vs almost always, often, or unsure) and daily smoking or use of other tobacco products. Information on the use of e-services comprised perceived competence (low or no vs moderate, high, or very high); perceived barriers and concerns (average of 6 items related to diverse challenges in using e-services); and perceived benefits (average of 6 items related to diverse benefits of electronic social and health care services). The scoring of the variables reflecting perceived cognitive function, barriers or concerns, and benefits of e-services followed principles used in related research [42,43] and ranged from 1 to 5, with greater values reflecting better cognitive function, greater barriers or concerns, and greater perceived benefits. For original questionnaire items, see Multimedia Appendix 1.

#### App Use

Data on the use of the app accumulated automatically throughout the study via log files that included time-stamped user interactions with the app. App log data were considered from completion of the baseline questionnaire to the completion of the questionnaire at the end of the follow-up or, if not completed, 90 days since the baseline. The app use metrics used in the effects assessment were the percentage of use days, the total number of reported habit performances, and the number of reported habit performances targeting each primary outcome (diet quality, physical activity, and BMI; Table 1). Similar use metrics that are based on logins and activities performed have been found to be positively associated with favorable changes in behavioral and anthropometric outcomes in digital lifestyle interventions [44], including the StopDia study [45]. Reported performances related to eating or drinking were considered to target diet quality, performances related to physical activity or sedentary behavior were considered to target total physical activity, and performances related to eating, drinking, physical activity, or sedentary behavior were considered to target BMI. In addition, to describe the overall app use over the follow-up, we computed the percentage of active users per week, the number of use days, the number of habit categories with reported performances, and the number of specific habits with reported performances (Table 1).

**Table 1.** Use metrics derived from the app log files.

Use metric	Definition
Active users (% per week)	Users with ≥1 login/all users×100
Use days	Number of days with logins
% Use days	Number of days with logins/number of follow-up days×100
N categories with performances	Number of habit categories with reported performances
N habits with performances	Number of specific habits with reported performances
N performances^a^	Number of reported habit performances
Total	Total
Targeting diet quality	Related to eating or drinking
Targeting physical activity	Related to physical activity or sedentary behavior
Targeting BMI	Related to eating, drinking, physical activity, or sedentary behavior

a A specific habit could be marked as performed a maximum of once per day.

#### Primary Outcomes

The primary outcomes comprised self-reported diet quality, total physical activity, and BMI. These data were collected with questionnaires via the app at the start of the follow-up (defined as the completion of the baseline questionnaire), midway through the follow-up (approximately 45 days since the baseline), and at the end of the follow-up (approximately 90 days since the baseline). Total physical activity and BMI were additionally measured in the questionnaire of the Healthy Finland survey that was completed pre-baseline [28], and for comparability, we used the same questionnaire items (Multimedia Appendix 1).

Diet quality was measured with the Healthy Diet Index (HDI) [46], which reflects adherence to the Nordic and Finnish food-based dietary guidelines on a scale ranging from 0 (lowest diet quality) to 100 (highest diet quality). The HDI builds on a food frequency questionnaire that covers 7 dietary domains (meal pattern; grains; fruit and vegetables; fats; fish and meat; dairy; and snacks and treats, including beverages) [46]. Total physical activity covered weekly hours of low-, moderate-, and high-intensity physical exertion. To capture potential changes in both the amount and intensity of total physical activity per week, the time spent at each intensity level was weighted with its approximate metabolic equivalent of task (MET): 2.5 for low-intensity, 5 for moderate-intensity, and 10 for high-intensity exertion. The cutoffs were determined based on the description of each intensity level in the questionnaire items, corresponding MET values reported in the literature [47,48], and consultation with an expert on sports science. The weighted estimates were totaled to reach total MET hours per week. With this weighting, 12.5 MET hours per week met the current recommendation for adults to accumulate at least 2.5 hours of moderate-intensity or 1.25 hours of high-intensity aerobic physical activity per week, or an equivalent combination of both [49,50]. BMI (kg/m^2^) was computed based on self-reported height and weight. While self-reported BMI is generally lower than measured BMI due to the underestimation of weight and overestimation of height, self-reported BMI tends to correlate with measured BMI over 90% and is considered a valid measure across a range of sociodemographic groups [51-53].

#### App Use Experience

User experiences were collected with a questionnaire via the app at the end of the follow-up. Questionnaire items reflected the acceptability and overall evaluation of the app (for original questionnaire items, see Multimedia Appendix 1). The mean acceptance of the app was computed by averaging the ratings (scale: 1‐4, higher value indicating greater acceptance) the participants gave to 7 questionnaire items that reflected the 7 component constructs of the Theoretical Framework of Acceptability [54]. These constructs comprise affective attitude (how the participant feels about taking part in the intervention), burden (the perceived amount of effort that is required to participate in the intervention), perceived effectiveness (the extent to which the intervention is perceived as likely to achieve its purpose), opportunity costs (the extent to which benefits, profits, or values must be given up to engage in the intervention), intervention coherence (the extent to which the participant understands the intervention and how the intervention works), self-efficacy (the participant’s confidence that they can perform the behaviors required to participate in the intervention), and ethicality (the extent to which the intervention has a good fit with the participant’s value system) [54].

The overall evaluation of the app was computed by averaging the ratings (scale: 0‐10, higher value indicating a more positive evaluation) the participants gave to 4 questionnaire items that asked them to evaluate the behavioral suggestions that the app offered, the app as a whole, the likelihood to use the app in the future, and the likelihood to recommend the app to a friend or a colleague. The latter item was also used to compute the net promoter score by categorizing the participants into detractors (rating ≤6), passively satisfied (rating=7‐8), and promoters (rating ≥9), and then subtracting the percentage of detractors from the percentage of promoters [55].

### Statistical Analyses

The effects assessment included participants who completed the baseline questionnaire and at least one questionnaire thereafter; that is, the questionnaire midway through and/or at the end of the follow-up. We call these participants the “completers” of the study. Linear mixed effects models were used to estimate (1) the associations between app use and the slopes of the primary outcomes (diet quality, total physical activity, and BMI), that is, the interaction of elapsed time and an app use metric; and (2) the modification of these associations by the participants’ baseline characteristics. The app use metrics were evaluated at the time of the completion of each questionnaire. The models related to diet quality and BMI were fitted with individual-level random intercepts and slopes. The models related to total physical activity were fitted with individual-level random intercepts only, as the inclusion of random slopes resulted in model convergence issues.

Separate models were fitted for each outcome–app use metric pair. The app use metrics reflecting the number of reported performances were log-transformed. The model coefficients corresponding to the log-transformed numbers of performances thus reflect the expected difference in the outcome corresponding to a 2.7-fold increase in the number of performances. All models were adjusted for the following baseline characteristics potentially influencing the app use and its effects [20,23,25,29]: age, sex, education, perceived health status, BMI, perceived cognitive function, diet quality, and total physical activity, as well as the competence, barriers or concerns, and perceived benefits of using e-services. Effect modification by the baseline characteristics was assessed by estimating the 3-way interaction parameters between each characteristic, app use metric, and elapsed time. The 3-way interaction reflected how the characteristic changed the association between the use metric and the slope of the outcome.

Regarding total physical activity, values >112 hours per week (ie, on average <8 hours per day for sedentary activities and rest) were excluded as implausible. Regarding diet quality and BMI, no implausible values were detected. To assess the sensitivity against large changes in the primary outcomes due to possible misreporting, the main models were fitted also by removing individuals with the largest changes reported between 2 successive measurements. The changes between successive measurements were standardized (ie, by subtracting the mean and dividing by the SD), and the sensitivity analyses were conducted by first removing individuals with the largest standardized change >5, and then those with the largest standardized change >3.

Analyses were performed with the R software (version 4.5.0; R Foundation for Statistical Computing [56]), using the package *lme4* [57] to fit the linear mixed effects models. Statistical significance was assessed with a likelihood ratio test, with a predetermined α level of .05. Data points with missing values were excluded from the analyses, and the data were assumed to be missing at random and that loss to follow-up was noninformative. As the analyses were of an exploratory nature, corrections for an increased type I error rate were not applied.

## Results

### Participant Characteristics

**Figure 2.**
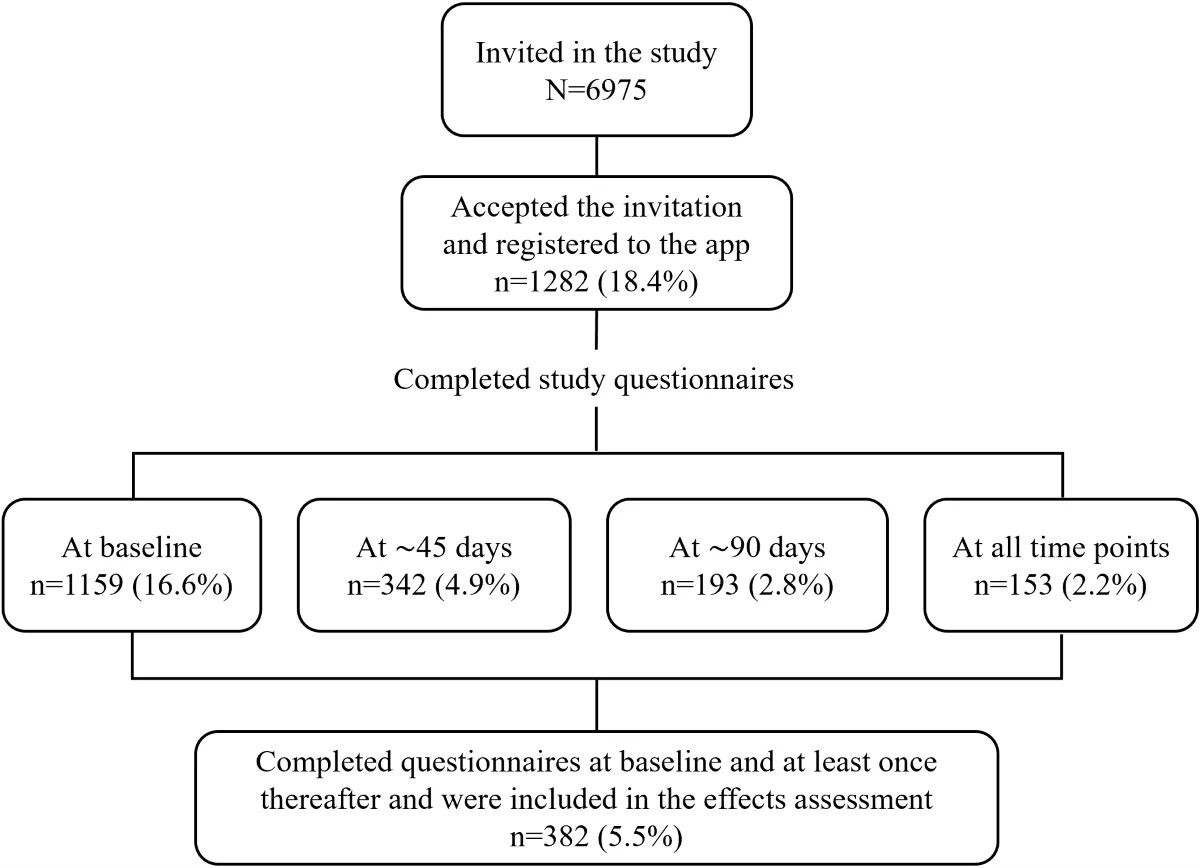
Formation of the study sample from invitees to completers (% invitees).

Of the 1282 individuals who accepted the study invitation and registered for the app (18.4% of invitees), 1159 (16.6%) completed the baseline questionnaire and 382 (5.5%) completed at least one questionnaire thereafter (“the completers”; Figure 2). The reasons for noncompletion are not available.

Among the completers, the proportion of men was 31% (119/382), age ranged from 20 to 74 (mean 51.3, SD 15.4) years, and the duration of full-time education ranged from 3 to 25 (mean 14.9, SD 3.4) years (Table 2). Most completers (280/382, 73%) belonged to the 3 higher income categories (household gross income approximately €2916‐€4580, or US $2953-US $4639, per month or more), and the majority were either employed or studying (218/382, 57%) or retired (134/382, 35%). A minority reported poor perceived health status (27/382, 7%), insufficient sleep (80/382, 21%), daily use of tobacco products (25/382, 7%), or poor competence to use e-services (7/382, 2%). On a scale ranging from 1 (poorer) to 5 (greater), the completers perceived their cognitive function to be on average 4.0 (SD 0.7), the extent to which they experienced barriers or concerns related to using e-services was on average 2.2 (SD 0.7), and the extent to which they perceived a benefit from e-services was on average 3.95 (SD 0.7).

**Table 2.** Participant characteristics at baseline.

Characteristic	Completers (n=382)^a^	Noncompleters (n=777)^b^	*P *value^c^
Sociodemographic characteristics			
Age (y), mean (SD)	51.3 (15.4)	49.9 (15.2)	.11
Sex (female), n (%)	263 (69)	516 (66)	.44
Education (y), mean (SD)	14.9 (3.4)	15.5 (3.28)	.004
Household income^d^ (per month), n (%)			.67
<€1250 (<US $1266)	30 (8)	55 (7)	
€1251‐€2915 (US $1267-US $2952)	69 (18)	157 (20)	
€2916‐€4580 (US $2953-US $4639)	120 (32)	222 (29)	
€4581‐€6250 (US $4640-US $6330)	83 (22)	162 (21)	
>€6251 (>US $6331)	77 (20)	176 (23)	
Principal life situation, n (%)			.03
Employed or studying	218 (57)	497 (64)	
Family leave or stay-at-home parent	4 (1)	18 (2)	
Retired	134 (35)	206 (27)	
Unemployed	14 (4)	31 (4)	
Other	12 (3)	20 (3)	
Health			
Perceived health status (fairly poor or poor), n (%)	27 (7)	51 (7)	.85
BMI (kg/m^2^), mean (SD)	27.6 (5.3)	27.9 (5.7)	.67
Perceived cognitive function (scale: 1‐5), mean (SD)	4.0 (0.7)	4.0 (0.7)	.55
Lifestyles			
Healthy Diet Index (scale: 0‐100), mean (SD)	54.4 (11.0)	52.5 (10.7)	.005
Total physical activity (MET^e^ hours per week), median (IQR)	30.0 (15.0‐50.0)	30.0 (16.1‐52.5)	.55
Enough sleep (rarely or hardly ever), n (%)	80 (21)	166 (21)	.98
Daily use of tobacco products, n (%)	25 (7)	64 (8)	.39
e-Service use			
Competence (low or no), n (%)	7 (2)	26 (3)	.21
Barriers and concerns (scale: 1‐5), mean (SD)	2.20 (0.73)	2.20 (0.74)	.86
Perceived benefits (scale: 1‐5), mean (SD)	3.95 (0.74)	3.89 (0.80)	.44

a Missing data: 0%‐2%.

b Missing data: 0%‐1%.

c Chi-square test for categorical and Mann-Whitney *U* test for continuous variables.

d Using a conversion rate of €1=US $1.0128, which was the average exchange rate during the data collection between September and December 2022.

e MET: metabolic equivalent of task.

Compared to the noncompleters (n=777), the completers had, on average, fewer years of education (14.9 vs 15.5 years; *P*=.004) and better diet quality (HDI 54.4 vs 52.5; *P*=.005; Table 2). The completers also differed from the noncompleters in their principal life situation (*P*=.03), with a greater proportion being retired (35% vs 27%) and a smaller proportion being employed or studying (57% vs 64%).

### App Use

**Figure 3.**
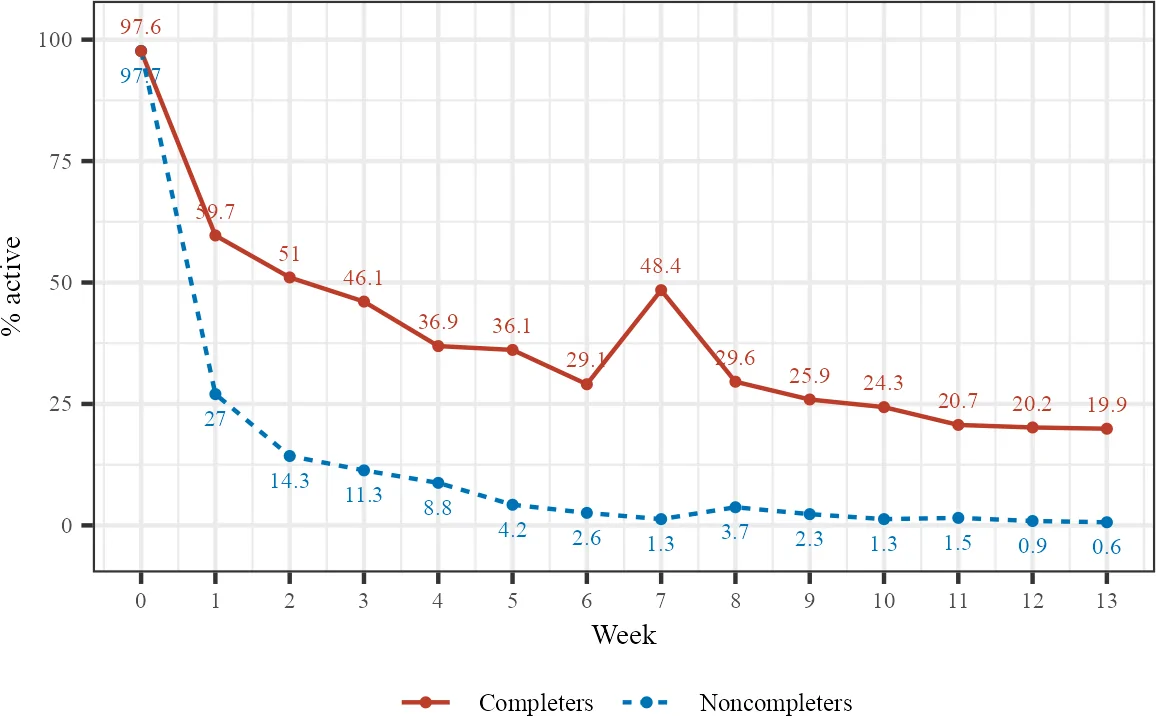
Percentage of active app users (≥1 login) per week over the 90-day follow-up.

Among the completers, the percentage of active app users dropped from 97.6% (373/382) at the beginning of the follow-up to 29.1% (111/382) by the 6th week of the follow-up and to 19.9% (76/382) by the 13th week of the follow-up (Figure 3).

Over the entire follow-up, the completers used the app on a median of 5.5 (IQR 3‐10) days (Table 3). This equaled a median of 6% (IQR 3%‐11%) of the follow-up days. The completers reported performances in altogether 14 (74%) of the 19 habit categories. These 14 categories were related to physical activity (aerobic exercise, muscle strengthening exercise, mobility and balance activities, and stretching one’s legs), eating and drinking (alcohol, mindful eating, drinking water, sugar, fruits and vegetables, dietary fats, salt, and meal pattern), sleep, and positive mood. The median number of habit categories with reported performances was 2 (IQR 1‐3), while the median number of specific habits with reported performances was 5 (IQR 2‐7). The median number of total reported performances was 22 (IQR 3‐68.5). Across the app use metrics examined, the app use was significantly more frequent among the completers than among the noncompleters (*P* values <.001).

**Table 3.** App use during the 90-day follow-up.

App use metric	Completers (n=382), median (IQR)	Noncompleters (n=777), median (IQR)	*P* value^a^
Use days	5.5 (3-10)	1 (1-2)	<.001
% Use days	5.9 (3.3‐11)	1.1 (1.1‐2.2)	<.001
N categories with performances	2 (1-3)	0 (0‐1)	<.001
N habits with performances	5 (2-7)	0 (0‐2)	<.001
N performances			
Total	22 (3‐68.5)	0 (0‐2)	<.001
Targeting diet quality	3 (0‐19)	0 (0‐0)	<.001
Targeting physical activity	4 (0‐25.75)	0 (0‐0)	<.001
Targeting BMI	13 (2‐49)	0 (0‐1)	<.001

a Mann-Whitney *U* test.

### Effect of App Use on Changes in Primary Outcomes

The completers’ mean diet quality was 54.4 (SD 11) HDI points out of 100 at baseline, rose by 1.7 points to 56.1 (SD 10.8) by the midpoint of the follow-up, and dropped by 1.5 points to 54.6 (SD 9.8) by the end of the follow-up (Table S1 in Multimedia Appendix 1). The median total physical activity was 30 (IQR 15‐50) MET hours per week at baseline, decreased by 1.25 units to 28.75 (IQR 17.5‐50) by the midpoint, and returned to 30 (IQR 17.5‐48.75) by the end of the follow-up. The mean BMI was 27.6 (SD 5.3) kg/m^2^ at baseline, decreased by 0.1 units to 27.5 (SD 5.2) kg/m^2^ by the midpoint, and returned to 27.6 (SD 5.7) kg/m^2^ by the end of the follow-up.

When estimating the effect of app use on changes in the primary outcomes over the 90-day follow-up, the results differed depending on the app use metric and the outcome. A higher percentage of app use days (by 10 percentage points) was associated with a 3.09 (95% CI 0.79‐5.39) MET hours per week greater change in total physical activity (Table 4). The percentage of use days was, however, not significantly associated with changes in diet quality or BMI. In contrast, a 1-unit higher logarithmic number of total reported habit performances (ie, 2.7-fold higher on the absolute scale) was associated with a 0.51 (95% CI 0.07-0.95) point greater change in diet quality. The total number of reported performances was, however, not significantly associated with changes in total physical activity or BMI. No significant associations were found between the number of reported performances targeting each outcome and the changes observed in the respective outcomes. For unadjusted effect estimates, see Table S2 in Multimedia Appendix 1.

**Table 4.** Effect of app use on changes in primary outcomes.

App use metric	Primary outcomes^a^					
	HDI (n=368)		TPA (n=362)		BMI (n=368)	
	β (95% CI)	*P* value	β (95% CI)	*P* value	β (95% CI)	*P* value
% Use days	.58 (–0.07 to 1.22)	.08	3.09 (0.79 to 5.39)	.01	.00 (–0.09 to 0.08)	.95
N performances^b^						
Total	.51 (0.07 to 0.95)	.02	1.30 (–0.30 to 2.89)	.11	–0.01 (–0.07 to 0.04)	.64
Targeting the outcome	.16 (–0.34 to 0.65)	.54	.62 (–1.02 to 2.2)	.47	–0.03 (–0.08 to 0.03)	.40

a Parameter estimates (β), 95% CI, and significance (*P*) of the effect of app use on Healthy Diet Index (HDI), total physical activity (TPA), and BMI. The estimates describe how changes in the outcomes over 90 days differ corresponding to a unit difference in the app use metric.

b Log-transformed.

The results remained predominantly robust in sensitivity analyses (Table S3 in Multimedia Appendix 1). The most notable changes were a slight attenuation of the estimated effect of the percentage of use days on physical activity (β=2.33, 95% CI 0.20-4.44; *P*=.03) and a slight strengthening of the estimated effect of the total number of performances on physical activity (β=1.71, 95% CI 0.29-3.10; *P*=.02) when removing individuals with the largest standardized change >3. In addition, the effect estimate for each app use metric on BMI was slightly stronger under the sensitivity analyses.

### Effect Modification by Baseline Characteristics

Greater physical activity at baseline appeared to enhance the effect of the app use on total physical activity regardless of the app use metric used: the percentage of app use days (β=.13, 95% CI 0.06-0.2), the total number of reported performances (β=.05, 95% CI 0.01-0.10), or the number of reported performances targeting physical activity (β=.05, 95% CI 0.00-0.09; Tables S4-S6 in Multimedia Appendix 1). This would mean that, in increasing movement, individuals who are physically more active at baseline benefit from using the app more than individuals who are physically less active.

Additionally, a greater extent to which the participants perceived to benefit from using e-services at baseline appeared to enhance the effect of the percentage of app use days (β=4.34, 95% CI 1.11-7.60) and the number of reported performances targeting physical activity (β=2.13, 95% CI 0.10-4.13) on total physical activity (Tables S4 and S6 in Multimedia Appendix 1). This would mean that, in increasing movement, individuals with more positive attitudes toward e-services at baseline benefit from using the app more than individuals with less positive attitudes. No baseline characteristic was found to modify the effect of the app use on diet quality or BMI (Tables S4-S6 in Multimedia Appendix 1).

### App Use Experience

The participants who completed the user experience questionnaire at the end of the study gave the app a median overall acceptability score of 2.9 (IQR 2.4‐3.3) on a scale ranging from 1 to 4, where a higher value indicated greater acceptance (Table 5). The participants gave both the app and the behaviors the app suggested a median overall score of 7 (IQRs 5‐8 and 6‐8, respectively) on a scale ranging from 0 to 10. Both the median likelihood to use the app in the future and to recommend the app to others was 5 (IQR 1‐8).

**Table 5.** App use experiences at the end of the study (n=199).

User experience metric	Values
Acceptability (scale: 1‐4^a^), median (IQR)	
Affective attitude	3 (2-3)
Burden	3 (2-3)
Perceived effectiveness	3 (2-3)
Opportunity costs	3 (2-4)
Intervention coherence	3 (2‐3.5)
Self-efficacy	3 (2-3)
Ethicality	3 (3-4)
Mean of all acceptability domains	2.86 (2.43‐3.29)
Overall evaluation (scale: 0‐10), median (IQR)	
The behaviors the app suggested	7 (6-8)
The app as a whole	7 (5-8)
Likelihood to use the app in the future	5 (1-8)
Likelihood to recommend the app to a friend or colleague	5 (1-8)
Promoters (rating 9‐10), n (%)	30 (15)
Passively satisfied (rating 7‐8), n (%)	43 (22)
Detractors (rating 0‐6), n (%)	126 (63)
Net promoter score (% promoters−% detractors)	–48

a Measures informed by the Theoretical Framework of Acceptability [54].

## Discussion

### Principal Findings

We assessed the effects and effect modifiers of the use of a habit-based healthy lifestyle app on self-reported diet quality, total physical activity, and BMI in a subsample of population-based health survey participants in Finland. The assessment was exploratory and correlational in nature, building on a quasi-experimental 1-group pre-post intervention. Over 3 months, the study participants used the app on a median of 5.9% (IQR 3%-11%) of days and reported a median of 22 (IQR 3-68.5) total habit performances. The percentage of use days was associated with increased physical activity, and the number of reported performances was associated with improved diet. Some evidence also suggested that greater baseline physical activity and a positive attitude toward e-services could enhance the app use effect on physical activity. Overall, the app was accepted and received moderate ratings. However, the likelihood at which the participants considered using the app in the future and recommending the app to others indicated that there is room to improve the app. The study contributes to the scant evidence base of the effects and factors that influence the effects of digital lifestyle interventions that use a habit-based approach, in a broadly recruited sample of adults. Moreover, the work provides a real-world example of scaling up a digital lifestyle intervention previously studied among individuals at risk of chronic diseases to a more heterogeneous audience.

### Comparison With Prior Research

#### App Use

With a median of 5.5 (IQR 3‐10) objectively measured app use days (5.9%, IQR 3%‐11% of the follow-up period) and 22 (IQR 3‐68.5) self-reported habit performances accumulated during the 3-month follow-up, many study participants were unlikely to succeed in forming new habits, particularly because the habit performances were divided into a median of 5 diverse behaviors. The minor changes observed in self-reported diet quality, physical activity, and BMI seem hence credible. While limited evidence exists of the time and number of repetitions needed to reach habit automaticity, a median of 66 (IQR 39-102) days was reported in a pioneering study that tracked the habit formation process among university students who performed a self-selected health-promoting action once per day in response to a stable cue [34]. The time needed for the habit strength to peak varies substantially, however, as the trajectory of habit formation is highly idiographic and often nonlinear [16]. Between-participant variation has been reported to range from 18 to 254 days [34,58]. Despite this variability, prior habit formation interventions have adopted a 3-month duration, considering it a sufficient period for a habit to develop—at least with daily repetition of the target behavior [15,17].

The overall low app use in this study suggests that the app was insufficiently engaging for many participants and may have failed to support them in repeating the selected behaviors. A voluntary habit-based behavior change requires sustained motivation and self-control to repeat the target behavior sufficiently for a habit to form [12]. To achieve this, people should be supported particularly during the early phases of their habit formation attempts because at this point, the emerging habit strengthens most quickly, but people are most likely to give the change attempt up if unsatisfied with initial experiences [12]. Common ways to support the continuation of the habit formation process include emphasizing the importance of the new behavior and training people in the skills needed to act in the chosen setting, for example, by making detailed action plans for the intended behavior and coping plans to overcome unintended behaviors [12]. Ideally, the support would be responsive to initial experiences and, as needed, assist in refining the set goals and methods chosen to pursue them [12].

The percentage of active app users dropped more quickly, and the app use frequency remained lower overall in this study than in the 12-month T2D prevention trial, StopDia, in which the BitHabit app was used by adults at risk of developing T2D [18,19]. During the first 6 months of StopDia, the percentage of active app users remained above 50%, the app was used on a median of 23 (IQR 12‐42) days, and the users reported a median of 263 (IQR 60‐703) habit performances [19]. In comparison, the Top Tips habit-based weight loss app reached an average of 25 (SD 44) logins and 10 (SD 21) completed tips during a 3-month pilot trial among individuals with overweight or obesity [17]. These differences in app use might be explained by the different target populations of the studies. While our study targeted the general adult population, the individuals who volunteered to participate tended to be healthier and have healthier lifestyles than those who did not [29,30]. The participants may hence have lacked a need or motivation to change their lifestyles. In contrast, the participants of StopDia were recruited via a T2D risk-screening website based on their risk scores (the Finnish Diabetes Risk Score ≥12 points [59]). The screening may have raised the participants’ risk awareness and motivation to manage the risk by improving their lifestyles.

Supporting the interpretation related to risk awareness and motivation for lifestyle modification, digital physical activity interventions have been found to yield greater effects among at-risk and sick individuals than among healthy populations [7]. The interpretation also receives support from the Theoretical Domains Framework, an integrative framework that synthesized 33 theories and 128 theoretical constructs related to behavior change into 14 theoretical domains that facilitate explaining and predicting behavior [60,61]. According to the Theoretical Domains Framework, key determinants of behavior include knowledge, such as the awareness of personal susceptibility to develop a health condition and the severity of the condition, and beliefs about consequences, such as expectations related to the benefits of changing versus the risks of not changing behavior [61]. These determinants can influence other determinants, such as intentions (ie, conscious decisions to act), goals (eg, goal setting and prioritizing), and behavioral regulation (eg, action planning and self-monitoring) [61-64]. All these determinants reflect psychological capability and reflective motivation, which belong to the necessary conditions of behavior according to the COM-B (capability, opportunity, motivation—behavior) system [65].

Despite the generally low intervention engagement observed in this study, the variability in app use was substantial, particularly in the number of reported habit performances. The top quartile reported more than 68.5 total performances, which is a considerable effort. Similar variability was observed in StopDia [19,20], in which 11% of participants used the BitHabit app approximately twice per week and the most active 4% nearly daily throughout the 1-year intervention, reporting a median of 1433 and 4516 total performances, respectively [20]. These observations highlight the value of this study and the need for continued research efforts to identify the characteristics of the population segment that will find inexpensively scalable lifestyle apps intended for self-directed well-being and health promotion engaging and beneficial. Advancements in this research space will facilitate the allocation of more resource-intensive health services to individuals who need them most. As one size does not fit all and no single intervention will solve the public health challenges societies face, we need various tools to serve various preferences and needs, together with knowledge on the types of individuals who benefit from each tool.

#### Effect of App Use on Changes in Primary Outcomes

The exploratory, quasi-experimental effects assessment was performed with 2 types of app use metrics that reflected participants’ engagement with the app: the percentage of app use days and the number of reported habit performances. Each metric had its pros and cons. The percentage of use days was a fully objective measure that was directly based on app logins and reflected the frequency with which the users visited the app. However, it did not reveal how the app was used during these visits. Habit performances, in contrast, reflected concrete actions taken for habit formation. They, however, were self-reported and could be scheduled in advance or recorded retrospectively, which made them subject to recall bias.

The associations observed between the app use and changes in the primary outcomes varied depending on the app use metric and the outcome. Together, however, the findings support an interpretation that improvements in diet quality and total physical activity were connected with the app use. Diet quality was statistically significantly associated with the total number of performances, and physical activity was associated with the percentage of app use days. In addition, the estimates for the associations of diet quality with the percentage of app use days and physical activity with the total number of performances were, albeit not statistically significant, in line with a favorable app use effect. These results suggest that an active use of a habit-promoting digital tool could facilitate small favorable changes in lifestyle habits. In contrast, no evidence was found for an association between BMI and app use. This might be due to the short duration of the follow-up and the fact that not all dietary or physical activity–related changes in behavior influence energy balance and thus body weight.

Interestingly, we observed no significant associations between changes in the primary outcomes and the number of reported habit performances specifically targeting each outcome. The only exception was a sensitivity analysis that found the performances related to BMI were associated with reduced BMI. While the number of actions that specifically advance an outcome could be considered a probable and precise predictor of improved outcomes, actions that are not directly related to the outcome can also contribute to its improvement. For example, actions that enhance mood or sleep can facilitate the repetition of diet-related habit performances, resulting in improved diet quality. This pathway could explain why we found evidence of an association between diet quality and the total number of habit performances, but not with diet-related performances.

Our results provide support for prior evidence that the effectiveness of a lifestyle app depends on its use. The StopDia trial demonstrated that a higher BitHabit user engagement—measured as at or above the median of 501 self-reported habit performances over the intervention year [18] or the number of monthly use days during intervention months 2 to 12 [20]—was associated with improvements in T2D risk factor levels, including diet quality, physical activity, and BMI. The greater the use was, the greater the benefits observed. While participants with sustained use once or twice per week showed small beneficial changes compared to those who terminated the use, participants with nearly daily use throughout the study showed the most beneficial changes [20].

Similarly, the habit-based Top Tips weight loss app that was piloted in a 3-month trial among adults with overweight or obesity was found most beneficial among active users [17]. Participants with the greatest changes in eating-related self-regulatory skills, weight, and adherence to target behaviors logged into the app and recorded their weight on average 2 to 3 times more and achieved the tips more often than participants with smaller changes in the outcomes [17]. Relatedly, a 4-week feasibility trial of an app that promoted vegetable intake with goal setting, self-monitoring, and feedback found the app use days and the frequency of recording vegetable intake via the app positively associated with increased vegetable intake among young adults [21].

The scientific evidence concerning the relationship between use and outcomes is not entirely consistent, however. Two systematic reviews examined this association in exclusively digital lifestyle interventions among adults [44,66]. One of them found a weak positive overall association between use and physical activity based on a meta-analysis of 11 studies and observed that the direction of the association was consistently positive for use metrics that reflected activities completed or logins [44]. The other review found mixed associations between use and dietary intake based on a narrative synthesis of 5 studies [66]. The inconsistency in the findings might be explained by, inter alia, differences in the digital lifestyle interventions and their target behaviors [66].

#### Effect Modification by Baseline Characteristics

We found some evidence that, in increasing movement, the use of the app might favor individuals who are physically more active and who have positive attitudes toward e-services. These characteristics also predicted the uptake of the app among the individuals who were invited to participate in this study [29,30]. The odds of uptake, though, were increased by a wide variety of individual characteristics, including middle age, female sex, higher education, higher income, better health, a healthier lifestyle (considering diet, physical activity, sleep, and smoking), as well as higher competence, fewer barriers, and greater perceived benefits of using e-services [29,30]. Hence, the study sample was not representative of the general adult population, which may have influenced the results of the effect modification analysis. In comparison, studies on the BitHabit app have found older age [20,22] and better diet quality [20] to predict greater engagement with the app.

Regarding lifestyle apps in general, a systematic review on factors influencing app adherence also reported that a positive attitude toward technology positively influences adherence to physical activity apps [23]. In contrast, this review found evidence of the positive influence of female gender on adherence to nutrition apps and of healthy weight on adherence to physical activity apps [23]. Another systematic review found overall mixed evidence of factors that influence the effectiveness of digital lifestyle interventions [25]. The review comprised 9 studies that examined inequality in effectiveness among adults with no medical conditions other than overweight or obesity. In these studies, greater effects were observed in participants with younger age (n=2 studies), older age (n=1), female gender (n=3), male gender (n=3), higher education (n=1), professional or managerial or executive job (n=1), and residence in the capital region (n=1) [25].

In sum, our preliminary, exploratory findings on the effect modifiers of the use of a habit-based app targeted at the general adult population—together with the evidence mentioned earlier of other digital lifestyle interventions—demonstrate the multitude of factors that may influence effects. The inconsistency in existing evidence warrants further efforts to identify groups that are and are not likely to benefit from digital lifestyle interventions. The inconsistency in findings across studies may be due to the heterogeneity of examined interventions, including but not limited to their target behaviors, theoretical underpinnings, approaches to support behavior change, components, content, intended use, target populations, evaluation context, and mode of delivery. Considering these characteristics could hence be meaningful in future studies that compare the effect modifiers of digital lifestyle interventions.

#### App Use Experience

The participants gave the app and the behaviors it suggested a median rating of 7 out of 10. Only 15% (30/199) were ready to promote the app to their friends or colleagues. The ratings indicate that the app failed to fully satisfy the needs of most participants. This might be because the behaviors included in the app library were rather general or because the app’s approach to personalization did not meet the users’ preferences. The personalization meant that the app determined and suggested habit categories with the greatest potential for improvement based on the user’s self-reported demographics, health behavior, perceived stress, and well-being goals. Digital lifestyle interventions have used varying approaches to personalization, but it remains unclear what type and level of personalization works and for whom [5]. For example, some may prefer a system-driven approach in which the digital tool determines the personalization, thus reducing the cognitive effort required from the user [5]. Some, in contrast, may prefer a user-driven approach that allows the user to control the personalization and thus provides a greater sense of autonomy [5]. For an increased degree of user-driven personalization, the app could, for example, allow and guide users to plan their own target habits and the contextual cues that are supposed to trigger their performance. This way, the pursued habits would better fit the individual’s lifestyle and daily routines and could thus strengthen their motivation and commitment to repeat the behaviors until they become automatic. These factors are considered crucial for the formation and maintenance of new habits [12,15,16].

### Strengths and Limitations

The strengths of the study include a behavioral theory–based digital lifestyle intervention, a web-based app that had been updated based on the learnings of a prior RCT among adults at risk of T2D [18]. In this study, the app was offered to a large group of adults sampled from the participants of a population-based health survey. As such, the study served as an attempt to scale the intervention up to a broader and more heterogeneous population. The study was rolled out in a realistic setting in which the app was simply made available for self-directed promotion of well-being and health, with no additional professional or peer support provided to boost its use. The assessments of the app use effect and effect modification incorporated participants’ objectively measured engagement with the app—a key determinant of effectiveness—and considered a wide variety of participant characteristics potentially influencing the effects. The work thus contributes to the scant evidence base on the effect modifiers of exclusively digital lifestyle interventions.

The results of the study must, however, be interpreted in the context of its limitations, particularly the lack of a control arm that precluded the assessment of causal effects and the reliance on self-reported data, both of which increase the uncertainty of our findings. To reduce the potential bias caused by self-reported data, we used measures that were validated (eg, the HDI), theory-based (the acceptability measures), and/or used in prior research (eg, the physical activity and BMI measurements); removed implausible responses from the data; and conducted sensitivity analyses that excluded the participants who reported the largest changes in the primary outcomes.

Further limitations include a multiplicity of analyses and outcomes, which increases the risk of type I error, and low participation and high attrition rates, which reduce the representativeness of our study sample to the general adult population and the statistical power to detect small effects. While the individuals to whom the app was offered represented a subsample of a population-based health survey that targeted a nationally representative random sample of adults [28], this subsample only included individuals who had chosen to participate in the survey by a certain date. Of these, 18% (1282/6975) accepted the offer and registered for the app. Of those registered, 30% (n=382) completed the measurements required for the effects assessment of this study. These individuals tended to be better off in terms of socioeconomic status, health, lifestyles, and e-service use. Our findings may thus have limited relevance to the broader adult population of Finland. Reaching and engaging more vulnerable population groups would likely require more tailored approaches.

The high attrition rate may cause bias if individuals who benefited from the app were more likely to complete the study questionnaires, leading to overoptimistic effect estimates. We compared the baseline characteristics of the completers and noncompleters of the study. The comparison showed that the completers had, on average, slightly fewer years of education and better diet quality and that they were more often retired than the noncompleters. The low participation and high attrition rate were not unusual, however, as they are widely reported challenges in digital lifestyle interventions [3,4,16,17,21], particularly when conducted among unselected target groups that may lack perceived need and motivation for lifestyle change [7,22]. In this study, the invitees had additionally recently participated in the population-based health survey, which may have reduced their motivation and resources to participate in this study as well. The findings of this study should therefore be considered preliminary, exploratory evidence that encourages further research in more robust study designs among more diverse and representative study populations with sufficient statistical power to detect small effects.

### Implications for Future Research

The findings of this study imply that for the development of more widely engaging habit-based digital lifestyle interventions, cocreation with the target group would be crucial. In addition, expert consensus supports a user-centered and iterative development that uses mixed methods and in-depth qualitative research to progressively refine the intervention to meet users’ context and needs [67]. Such work could also deepen our understanding of the relationship between the engagement with and the effects of the app [66], for example, by facilitating the characterization of “effective engagement”—which may not simply mean “more engagement”—to achieve the intended outcomes [67].

### Conclusions

This exploratory, correlational study suggests that an inexpensive, low-intensity digital support for healthy habits could foster small beneficial lifestyle changes if it succeeds in sustaining the user’s engagement. However, effects may depend on the individual. Identifying those most likely to benefit from such exclusively digital interventions calls for deeper insight into the modifiers of user engagement and effects.

## Supplementary material

10.2196/84076 Multimedia Appendix 1 Original questionnaire items and additional tables.

10.2196/84076 Checklist 1 STROBE checklist.

## Abbreviations

COM-B: capability, opportunity, motivation—behavior
HDI: Healthy Diet Index
MET: metabolic equivalent of task
RCT: randomized controlled trial
StopDia: Stop Diabetes
STROBE: Strengthening the Reporting of Observational Studies in Epidemiology
T2D: type 2 diabetes
