# Supplementary material for: The Usage Effects, Effect Modifiers, and Experiences of a Web-Based App for Healthy Habit Formation in Adults: Exploratory Analysis of a Quasi-Experimental Real-World Intervention
Source: JMIR Mhealth Uhealth. 2026 Sep 21;14:e84076. doi: 10.2196/84076 (PMC13592743; doi:10.2196/84076)
Supplement: Supplementary file 1 [file mhealth-v14-e84076-s001.docx]

This is a Multimedia Appendix to a full manuscript published in the JMIR Mhealth Uhealth. For full copyright and citation information, see: <https://doi.org/10.2196/84076>

**Content**

[1.	Original questionnaire items used in the study	2](#_Toc236657342)

[2.	Results	5](#_Toc236657343)

[Table S1. Primary outcome measures at various timepoints among the completers (n=382)	5](#_Toc236657344)

[Table S2. Unadjusted effects of app use on changes in primary outcomes	5](#_Toc236657345)

[Table S3. Sensitivity analyses of the effects of app use on changes in primary outcomes.	6](#_Toc236657346)

[Table S4. Effect modification of the percentage of app use days by baseline characteristics	7](#_Toc236657347)

[Table S5. Effect modification of the logarithmic number of total reported performances by baseline characteristics	8](#_Toc236657348)

[Table S6. Effect modification of the logarithmic number of reported performances targeting each outcome by baseline characteristics	9](#_Toc236657349)

# 1. Original questionnaire items used in the study

**Background information**

**Socioeconomics**

How many years altogether have you attended school or studied full time? *Including primary and comprehensive school.*

How large was your household's income last year (before taxes)?

- less than 15 000 € (less than 1250 €/month)
- 15 001 - 35 000 € (about 1251–2915 €/month)
- 35 001 - 55 000 € (about 2916–4580 €/month)
- 55 001 - 75 000 € (about 4581–6250 €/month)
- more than 75 000 € (6251 €/month or more)

At the moment, are you principally: *Please choose the option that best describes your situation*

- employed full-time
- employed part-time
- retired on an old age pension
- receiving a disability pension or rehabilitation benefit
- on part-time retirement
- unemployed or laid off
- on family leave or a stay-at-home mother/father
- a student or on study leave
- other

**Health**

How would you describe your state of health at present?

- good
- fairly good
- average
- fairly poor
- poor

How tall are you? _________ *cm, please round to nearest centimeter*

How much do you weigh when wearing light clothing? _________ *kg, please round to nearest kilogramme*

**Cognitive function**

How would you rate your memory and your ability to learn and concentrate? (very well, well, adequately, poorly, very poorly)

- how well does your memory work?
- how easily do you learn new things?
- how well can you concentrate on things?

**Lifestyle habits**

***Diet Quality***

Healthy Diet Index. For the food frequency questionnaire, please refer to: Lindström J, Aittola K, Pölönen A, Hemiö K, Ahonen K, Karhunen L, Männikkö R, Siljamäki-Ojansuu U, Tilles-Tirkkonen T, Virtanen E, Pihlajamäki J, Schwab U. Formation and Validation of the Healthy Diet Index (HDI) for Evaluation of Diet Quality in Healthcare. Int J Environ Res Public Health 2021;18:2362. doi: 10.3390/ijerph18052362.

***Physical Activity***

How much exercise do you get in a week in the course of your work, commute, and spare time (total hours and minutes per week)? Think about the past 12 months. Take all regular, weekly physical exertion in consideration.

- hardly any regular exercise
- low-intensity aerobic exercise (= does not make you sweat or get out of breath, e.g. walking leisurely)
- moderate-intensity aerobic exercise (= makes you sweat a bit and/or get slightly out of breath, e.g. walking briskly)
- high-intensity aerobic exercise (= makes you sweat a lot and/or get out of breath, e.g. jogging or running)

***Sleep***

Do you feel that you get enough sleep?

- yes, almost always
- yes, often
- rarely or hardly ever
- not sure

***Smoking or use of other tobacco products***

Do you smoke currently (cigarettes, cigars, or pipe)?

- yes, daily
- occasionally
- not at all
- I have never smoked

Do you currently use any of the following products? (yes, daily; yes, occasionally; not at all; I have never used)

- snus (Swedish type moist snuff)
- e-cigarettes with nicotine
- e-cigarettes without nicotine
- nicotine replacement therapy products such as patches or chewing gum
- heated tobacco products
- nicotine pouches

**E-service use**

***Competence***

How would you rate your competence to use online services (on a computer or smartphone)?

- no competence
- low
- moderate
- high
- very high

***Perceived barriers and concerns***

How do you feel about the following statements about electronic services? (completely agree, somewhat agree, neither agree nor disagree, somewhat disagree, strongly disagree)

- face-to-face encounters cannot be replaced by electronic contacts
- the electronic services are not accessible to me, e.g., due to my visual impairment
- the services I need are not available electronically
- I am concerned about data security when it comes to my personal details
- data connections are poor in my area
- I need help with using the online services

***Perceived benefits***

How do you feel about the following statements concerning the benefits of electronic social and health care services? (completely agree, somewhat agree, neither agree nor disagree, somewhat disagree, strongly disagree)

Electronic services…

- help me to assess the need for services
- support me in finding and choosing the most suitable services
- make it easier for me to use services regardless of where I am and when
- make it easier for me to collaborate with professionals
- help me to take an active role in looking after my own health and welfare
- help me to take care of the health, welfare and functional capacity of family or friends

**User experience**

**Acceptability (measures informed by the Theoretical Framework of Acceptability** ^1^**)**

(1=completely disagree, 2= somewhat disagree, 3=somewhat agree, 4=completely agree)

[Affective attitude] Does using the app make you feel good?

[Burden] Is using the app easy and effortless?

[Perceived effectiveness] Can using the app help you live a healthier life?

[Opportunity costs] Does using the app take too much of your time or energy? ^2^

[Intervention coherence] Is it easy to understand how using the app relates to establishing healthy lifestyles?

[Self-efficacy] Do you trust that you can perform the small actions offered by the app?

[Ethicality] Do the small actions offered by the app align with what you consider important in your life?

^1^ Sekhon M, Cartwright M, Francis JJ. Acceptability of healthcare interventions: an overview of reviews and development of a theoretical framework. *BMC Health Serv Res*. 2017;17:88. doi:10.1186/s12913-017-2031-8

^2^ Coding reversed for analysis: 1=completely agree, 4=completely disagree

**Overall evaluation**

What rating would you give to the small actions offered by the app? (0=worst, 10=best)

What rating would you give to the app as a whole? (0=worst, 10=best)

How likely are you to use the app in the future? (0=unlikely, 10=likely)

How likely are you to recommend the app to a friend or colleague? (0=unlikely, 10=likely)

# 3. Results

## Table S1. Primary outcome measures at various timepoints among the completers (n=382)

| Outcome variable | Pre-Baseline^a^ | Baseline^b^ | Midpoint^c^ | Post^d^ |
| --- | --- | --- | --- | --- |
| HDI^e^, mean (SD) | Not available | 54.4 (11) | 56.1 (10.8) | 54.6 (9.8) |
| TPA^f^, median (IQR) | 35 (17.5–56.6) | 30 (15–50) | 28.75 (17.5–50) | 30 (17.5–48.75) |
| BMI, mean (SD) | 27.4 (5.3) | 27.6 (5.3) | 27.5 (5.2) | 27.6 (5.7) |

^a^Missing data: 0–0.3%. Data collected in the Healthy Finland survey a median of 148 (range 47–231) days before the baseline of this study.

^b^Missing data: 0–0.3%.

^c^Missing data: 10.5–11%

^d^Missing data: 49.5%

^e^Healthy Diet Index, scale: 0–100

^f^Total physical activity, MET-hours/week

## Table S2. Unadjusted effects of app use on changes in primary outcomes

| App use metric | | Primary outcomes^a^ | | | | | |
| --- | --- | --- | --- | --- | --- | --- | --- |
|  | | HDI (n=382) | | TPA (n=374) | | BMI (n=380) | |
|  | | β (95% CI) | *P* | β (95% CI) | *P* | β (95% CI) | *P* |
| % Use days | | 0.80 (0.14 to 1.46) | .02 | 2.83 (0.52 to 5.14) | .02 | –0.01 (–0.09 to 0.08) | .89 |
| n Performances^b^ | |  |  |  |  |  |  |
|  | Total | 0.60 (0.15 to 1.04) | .01 | 1.34 (–0.26 to 2.93) | .10 | –0.02 (–0.07 to 0.04) | .58 |
|  | Targeting the outcome | 0.29 (–0.21 to 0.78) | .26 | 0.83 (–0.78 to 2.44) | .31 | –0.03 (–0.08 to 0.03) | .35 |

^a^Parameter estimates (β), 95% confidence intervals (CI), and significance (*P*) of the effect of app use on Healthy Diet Index (HDI), total physical activity (TPA), and BMI. The estimates describe how changes in the outcomes over 90 days differ corresponding to a unit difference in the app use metric.

^b^Logarithm

## Table S3. Sensitivity analyses of the effects of app use on changes in primary outcomes.

| App use metric | | | Primary outcomes^a^ | | | | | | | | |
| --- | --- | --- | --- | --- | --- | --- | --- | --- | --- | --- | --- |
|  | | | HDI | | | TPA | | | BMI | | |
|  | | | n | β (95% CI) | *P* | n | β (95% CI) | *P* | n | β (95% CI) | *P* |
| % Use days | | |  |  |  |  |  |  |  |  |  |
|  | z-score≤5 | | 368 | 0.58 (–0.07 to 1.22) | .08 | 361 | 2.96 (0.71 to 5.21) | .01 | 365 | –0.03 (–0.10 to 0.05) | .48 |
|  | z-score≤3 | | 366 | 0.61 (–0.03 to 1.24) | .06 | 349 | 2.33 (0.20 to 4.44) | .03 | 363 | –0.02 (–0.09 to 0.05) | .58 |
| N Performances^b^ | | |  |  |  |  |  |  |  |  |  |
|  | Total | |  |  |  |  |  |  |  |  |  |
|  |  | z-score≤5 | 368 | 0.51 (0.07 to 0.95) | .02 | 361 | 1.12 (–0.47 to 2.68) | .17 | 365 | –0.04 (–0.09 to 0.01) | .13 |
|  |  | z-score≤3 | 366 | 0.54 (0.11 to 0.98) | .02 | 349 | 1.71 (0.29 to 3.10) | .02 | 363 | –0.04 (–0.09 to 0.01) | .09 |
|  | Targeting the outcome | |  |  |  |  |  |  |  |  |  |
|  |  | z-score≤5 | 368 | 0.16 (–0.34 to 0.65) | .54 | 361 | 0.36 (–1.27 to 1.91) | .69 | 365 | –0.05 (–0.10 to 0.00) | .05 |
|  |  | z-score≤3 | 366 | 0.18 (–0.32 to 0.66) | .48 | 349 | 0.89 (–0.58 to 2.29) | .24 | 363 | –0.06 (–0.11 to –0.01) | .03 |

^a^Parameter estimates (β), 95% confidence intervals (CI), and significance (*P*) of the effect of app use on Healthy Diet Index (HDI), total physical activity (TPA), and BMI; excluding individuals whose largest standardized change between two successive measurements (ie, z-score) was >5 and >3. The estimates describe how changes in the outcomes over 90 days differ corresponding to a unit difference in the app use metric.

**^b^**Logarithm

## Table S4. Effect modification of the percentage of app use days by baseline characteristics

| Effect modifier | | | Primary outcomes^a^ | | | | | |
| --- | --- | --- | --- | --- | --- | --- | --- | --- |
|  | | | HDI | | TPA | | BMI | |
|  | | | β (95% CI) | *P* | β (95% CI) | *P* | β (95% CI) | *P* |
| Sociodemographic characteristics | | |  |  |  |  |  |  |
|  | Age, years | | 0.02 (–0.01 to 0.06) | .20 | 0.02 (–0.11 to 0.15) | .75 | –0.02 (–0.06 to 0.03) | .49 |
|  | Sex, female | | 0.06 (–1.98 to 2.01) | .99 | 1.67 (–5.62 to 8.91) | .66 | –0.07 (–0.32 to 0.18) | .59 |
|  | Education, years | | 0.03 (–0.13 to 0.20) | .69 | –0.40 (–2.4 to 1.59) | .69 | –0.01 (–0.03 to 0.01) | .48 |
| Health | | |  |  |  |  |  |  |
|  | Perceived health status, fairly poor or poor | | 1.71 (–2.00 to 5.39) | .37 | 2.32 (–11.62 to 16.2) | .75 | –0.35 (–0.78 to 0.08) | .12 |
|  | Body mass index | |  | .75 |  | .31 |  | .67 |
|  |  | < 25 | Reference |  | Reference |  | Reference |  |
|  |  | ≥ 25 to < 30 | –0.16 (–1.61 to 1.26) |  | –0.97 (–6.12 to 4.21) |  | 0.06 (–0.13 to 0.25) |  |
|  |  | ≥ 30 | 0.60 (–1.24 to 2.40) |  | –5.07 (–11.63 to 1.44) |  | –0.05 (–0.28 to 0.19) |  |
|  | Perceived cognitive function | | 0.58 (–0.39 to 1.53) | .24 | –1.82 (–5.34 to 1.73) | .32 | 0.07 (–0.05 to 0.19) | .27 |
| Lifestyles | | |  |  |  |  |  |  |
|  | Healthy Diet Index | | 0.03 (–0.03 to 0.09) | .28 | 0.12 (–0.09 to 0.33) | .27 | –0.04 (–0.12 to 0.04) | .30 |
|  | Total physical activity | | –0.01 (–0.20 to 0.19) | .96 | 0.13 (0.06 to 0.20) | <.001 | –0.01 (–0.03 to 0.02) | .65 |
| e-Service use | | |  |  |  |  |  |  |
|  | Competence, low or no | | 3.83 (–5.80 to 13.43) | .44 | 13.96 (–21.29 to 48.79) | .44 | 0.10 (–1.23 to 1.42) | .89 |
|  | Barriers and concerns | | 0.40 (–0.53 to 1.33) | .40 | 3.30 (–0.04 to 6.69) | .05 | 0.00 (–0.12 to 0.12) | .98 |
|  | Perceived benefits | | 0.42 (–0.47 to 1.33) | .35 | 4.34 (1.11 to 7.60) | .01 | 0.02 (–0.10 to 0.14) | .80 |

^a^Parameter estimates (β), 95% confidence intervals (CI), and the significance (*P*) of the effect modification on Healthy Diet Index (HDI, n=368), total physical activity (TPA, n=362), and BMI (n=368).

## Table S5. Effect modification of the logarithmic number of total reported performances by baseline characteristics

| Effect modifier | | | Primary outcomes^a^ | | | | | |
| --- | --- | --- | --- | --- | --- | --- | --- | --- |
|  | | | HDI | | TPA | | BMI | |
|  |  |  | β (95% CI) | *P* | β (95% CI) | *P* | β (95% CI) | *P* |
| Sociodemographic characteristics | | |  |  |  |  |  |  |
|  | Age, years | | 0.01 (–0.01 to 0.04) | .31 | –0.07 (–0.18 to 0.03) | .18 | –0.01 (–0.05 to 0.03) | .58 |
|  | Sex, female | | –0.08 (–1.00 to 0.81) | .84 | –1.56 (–4.87 to 1.74) | .35 | 0.08 (–0.04 to 0.20) | .19 |
|  | Education, years | | 0.00 (–0.12 to 0.12) | .99 | –0.68 (–2.12 to 0.77) | .36 | 0.00 (–0.02 to 0.02) | .97 |
| Health | | |  |  |  |  |  |  |
|  | Perceived health status, fairly poor or poor | | –0.15 (–1.92 to 1.59) | .64 | 1.75 (–4.59 to 8.16) | .58 | 0.11 (–0.13 to 0.34) | .36 |
|  | Body mass index, kg/m^2^ | |  | .87 |  | .30 |  | .34 |
|  |  | < 25 | Reference |  | Reference |  | Reference |  |
|  |  | ≥ 25 to < 30 | –0.23 (–1.24 to 0.75) |  | 0.22 (–3.40 to 3.88) |  | 0.09 (–0.04 to 0.22) |  |
|  |  | ≥ 30 | –0.22 (–1.39 to 0.93) |  | –2.82 (–7.03 to 1.39) |  | 0.02 (–0.12 to 0.17) |  |
|  | Perceived cognitive function | | 0.29 (–0.37 to 0.95) | .39 | –0.80 (–3.19 to 1.67) | .54 | 0.06 (–0.02 to 0.15) | .16 |
| Lifestyles | | |  |  |  |  |  |  |
|  | Healthy Diet Index | | 0.01 (–0.03 to 0.05) | .68 | –0.12 (–0.28 to 0.03) | .12 | –0.01 (–0.06 to 0.04) | .73 |
|  | Total physical activity | | 0.02 (–0.10 to 0.14) | .76 | 0.05 (0.01 to 0.10) | .02 | 0.00 (–0.01 to 0.02) | .74 |
| e-Service use | | |  |  |  |  |  |  |
|  | Competence, low or no | | –0.52 (–3.30 to 2.28) | .72 | –0.67 (–10.34 to 8.91) | .89 | 0.10 (–0.27 to 0.48) | .59 |
|  | Barriers and concerns | | 0.21 (–0.41 to 0.82) | .51 | 1.36 (–0.90 to 3.64) | .24 | –0.03 (–0.11 to 0.05) | .49 |
|  | Perceived benefits | | 0.03 (–0.49 to 0.56) | .90 | 1.57 (–0.34 to 3.49) | .11 | –0.05 (–0.12 to 0.02) | .18 |

^a^Parameter estimates (β), 95% confidence intervals (CI), and the significance (*P*) of the effect modification on Healthy Diet Index (HDI, n=368), total physical activity (TPA, n=362), and BMI (n=368).

## Table S6. Effect modification of the logarithmic number of reported performances targeting each outcome by baseline characteristics

| Effect modifier | | | Primary outcomes^a^ |  |  |  |  |  |
| --- | --- | --- | --- | --- | --- | --- | --- | --- |
|  | | | HDI |  | TPA |  | BMI |  |
|  | | | β (95% CI) | *P* | β (95% CI) | *P* | β (95% CI) | *P* |
| Sociodemographic characteristics | | |  |  |  |  |  |  |
|  | Age, years | | 0.02 (–0.01 to 0.05) | .22 | –0.08 (–0.18 to 0.02) | .11 | –0.01 (–0.05 to 0.03) | .52 |
|  | Sex, female | | 0.41 (–0.63 to 1.42) | .45 | –0.57 (–3.96 to 2.84) | .75 | 0.06 (–0.06 to 0.18) | .33 |
|  | Education, years | | 0.00 (–0.15 to 0.15) | .98 | –0.38 (–1.85 to 1.10) | .62 | 0.00 (–0.02 to 0.01) | .90 |
| Health | | |  |  |  |  |  |  |
|  | Perceived health status, fairly poor or poor | | –0.36 (–2.20 to 1.46) | .68 | 2.29 (–6.28 to 10.99) | .59 | 0.15 (–0.10 to 0.39) | .25 |
|  | Body mass index (kg/m^2^) | |  | .81 |  | .13 |  | .67 |
|  |  | < 25 | Reference |  | Reference |  | Reference |  |
|  |  | ≥ 25 to < 30 | 0.36 (–0.76 to 1.49) |  | –2.04 (–5.72 to 1.65) |  | 0.05 (–0.08 to 0.19) |  |
|  |  | ≥ 30 | 0.22 (–1.08 to 1.51) |  | –4.40 (–8.62 to –0.16) |  | 0.00 (–0.15 to 0.15) |  |
|  | Perceived cognitive function | | 0.28 (–0.48 to 1.03) | .47 | –0.99 (–3.49 to 1.56) | .45 | 0.06 (–0.03 to 0.15) | .17 |
| Lifestyles | | |  |  |  |  |  |  |
|  | Healthy Diet Index | | 0.02 (–0.03 to 0.06) | .45 | –0.07 (–0.22 to 0.09) | .40 | –0.03 (–0.08 to 0.03) | .30 |
|  | Total physical activity | | –0.13 (–0.27 to 0.00) | .06 | 0.05 (0.00 to 0.09) | .06 | 0.01 (–0.01 to 0.02) | .35 |
| e-Service use | | |  |  |  |  |  |  |
|  | Competence, low or no | | –0.07 (–4.98 to 4.86) | .98 | –0.67 (–11.69 to 10.28) | .90 | 0.10 (–0.31 to 0.52) | .62 |
|  | Barriers and concerns | | –0.16 (–0.89 to 0.54) | .64 | 1.83 (–0.42 to 4.17) | .11 | –0.03 (–0.11 to 0.06) | .51 |
|  | Perceived benefits | | 0.05 (–0.59 to 0.70) | .86 | 2.13 (0.10 to 4.13) | .04 | –0.05 (–0.13 to 0.02) | .15 |

^a^Parameter estimates (β), 95% confidence intervals (CI), and the significance (*P*) of the effect modification on Healthy Diet Index (HDI, n=368), total physical activity (TPA, n=362), and BMI (n=368).
