# Supplementary material for: The Usage Effects, Effect Modifiers, and Experiences of a Web-Based App for Healthy Habit Formation in Adults: Exploratory Analysis of a Quasi-Experimental Real-World Intervention
Source: JMIR Mhealth Uhealth. 2026 Sep 21;14:e84076. doi: 10.2196/84076 (PMC13592743; doi:10.2196/84076)
Supplement: Supplementary file 2 [file mhealth-v14-e84076-s002.docx]

# STROBE Statement—Checklist of items that should be included in reports of *cohort studies*

|  | **Item No** | **Recommendation** | **Reported on manuscript section/subsection** |
| --- | --- | --- | --- |
| **Title and abstract** | 1 | (*a*) Indicate the study’s design with a commonly used term in the title or the abstract | (a) Title and Abstract  (b) Abstract |
|  |  | (*b*) Provide in the abstract an informative and balanced summary of what was done and what was found |  |
| **Introduction** | | | |
| Background/  rationale | 2 | Explain the scientific background and rationale for the investigation being reported | Introduction |
| Objectives | 3 | State specific objectives, including any prespecified hypotheses | Introduction, last paragraph |
| **Methods** | | | |
| Study design | 4 | Present key elements of study design early in the paper | Methods/Study Design |
| Setting | 5 | Describe the setting, locations, and relevant dates, including periods of recruitment, exposure, follow-up, and data collection | Methods/Study Design  Methods/Participant Recruitment |
| Participants | 6 | (*a*) Give the eligibility criteria, and the sources and methods of selection of participants. Describe methods of follow-up | (a) Methods/Study Design and  Participant Recruitment  (b) Not applicable |
|  |  | (*b*) For matched studies, give matching criteria and number of exposed and unexposed |  |
| Variables | 7 | Clearly define all outcomes, exposures, predictors, potential confounders, and effect modifiers. Give diagnostic criteria, if applicable | Methods/The App  Methods/Measures  Methods/Statistical Analyses  Multimedia Appendix/ Original questionnaire items used in the study |
| Data sources/  measurement | 8* | For each variable of interest, give sources of data and details of methods of assessment (measurement). Describe comparability of assessment methods if there is more than one group | Methods/Measures  Multimedia Appendix 1, Original questionnaire items used in the study |
| Bias | 9 | Describe any efforts to address potential sources of bias | Methods/Participant Recruitment   - Sampling protocol   Methods/Measures   - Validated, theory-based, and/or previously used self-report measures - Objective app usage measures that prior studies have found positively associated with favourable changes in behavioural and anthropometric outcomes.   Methods/Statistical Analyses   - Models adjusted with a range of covariates - Removal of implausible responses - Sensitivity analyses |
| Study size | 10 | Explain how the study size was arrived at | Methods/Participant Recruitment |
| Quantitative variables | 11 | Explain how quantitative variables were handled in the analyses. If applicable, describe which groupings were chosen and why | Methods/Measures  Methods/Statistical Analyses |
| Statistical methods | 12 | (*a*) Describe all statistical methods, including those used to control for confounding | Methods/Statistical Analyses |
|  |  | (*b*) Describe any methods used to examine subgroups and interactions |  |
|  |  | (*c*) Explain how missing data were addressed |  |
|  |  | (*d*) If applicable, explain how loss to follow-up was addressed |  |
|  |  | (*e*) Describe any sensitivity analyses |  |
| **Results** | | |  |
| Participants | 13* | (a) Report numbers of individuals at each stage of study—eg numbers potentially eligible, examined for eligibility, confirmed eligible, included in the study, completing follow-up, and analysed | Results/Participant Characteristics |
|  |  | (b) Give reasons for non-participation at each stage |  |
|  |  | (c) Consider use of a flow diagram |  |
| Descriptive data | 14* | (a) Give characteristics of study participants (eg demographic, clinical, social) and information on exposures and potential confounders | Results/Participant Characteristics |
|  |  | (b) Indicate number of participants with missing data for each variable of interest |  |
|  |  | (c) Summarise follow-up time (eg, average and total amount) |  |
| Outcome data | 15* | Report numbers of outcome events or summary measures over time | Results/Effect of App Use on Changes in Primary Outcomes  Multimedia Appendix 1, Table S1 |

| Main results | 16 | (*a*) Give unadjusted estimates and, if applicable, confounder-adjusted estimates and their precision (eg, 95% confidence interval). Make clear which confounders were adjusted for and why they were included | Adjusted: Results/Effect of App Use on Changes in Primary Outcomes, Table 4 (covariates included in the models explained in Methods/Statistical Analyses)  Unadjusted: Multimedia Appendix 1, Table S2 |
| --- | --- | --- | --- |
|  |  | (*b*) Report category boundaries when continuous variables were categorized |  |
|  |  | (*c*) If relevant, consider translating estimates of relative risk into absolute risk for a meaningful time period |  |
| Other analyses | 17 | Report other analyses done—eg analyses of subgroups and interactions, and sensitivity analyses | Results/Effect of App Use on Changes in Primary Outcomes  Results/Effect Modification by Baseline Characteristics  Results/App Use Experience  Multimedia Appendix 1, Tables S3–S6 |
| **Discussion** | | | |
| Key results | 18 | Summarise key results with reference to study objectives | Discussion/Principal Findings |
| Limitations | 19 | Discuss limitations of the study, taking into account sources of potential bias or imprecision. Discuss both direction and magnitude of any potential bias | Discussion/Strengths and Limitations |
| Interpretation | 20 | Give a cautious overall interpretation of results considering objectives, limitations, multiplicity of analyses, results from similar studies, and other relevant evidence | Discussion/Comparison With Prior Research  Discussion/Strengths and Limitations  Discussion/Conclusions |
| Generalisability | 21 | Discuss the generalisability (external validity) of the study results | Discussion/Strengths and Limitations |
| **Other information** | | | |
| Funding | 22 | Give the source of funding and the role of the funders for the present study and, if applicable, for the original study on which the present article is based | Funding |

*Give information separately for exposed and unexposed groups.

**Note:** An Explanation and Elaboration article discusses each checklist item and gives methodological background and published examples of transparent reporting. The STROBE checklist is best used in conjunction with this article (freely available on the Web sites of PLoS Medicine at http://www.plosmedicine.org/, Annals of Internal Medicine at http://www.annals.org/, and Epidemiology at http://www.epidem.com/). Information on the STROBE Initiative is available at http://www.strobe-statement.org.
